# A fast and cost-efficient method to detect ethanol as adulterant in gasoline

**DOI:** 10.1016/j.mex.2020.100974

**Published:** 2020-06-20

**Authors:** Adesina S. Fadairo, Joy Ekoh-Chukwukalu, Gbadegesin A. Adeyemi, Olusegun G. Abolarin, Isreal M.F. Mkpaoro

**Affiliations:** a Department of Petroleum Engineering, Covenant University Ota, Nigeria; b Schlumberger Technical Services Inc. Porto de Luanda, Angola; c University of Port Harcourt, Port Harcourt, Nigeria

**Keywords:** Petroleum products, Adulterated fuel, Potassium dichloromethane

## Abstract

Adulteration of fuel is a global problem that needs to be curbed. Studies have proved that no technically easy solution has been developed and easily accessible to the Oil and gas industry to detect adulterated gasoline. The need therefore arises for adequate and substantial methods for easy detection of adulterated gasoline.

• This study developed an effective and cost-efficient method to detect adulterated gasoline at the point of sales of Petroleum Product through the means of piece of litmus paper testing.
• The method provides the marketers and gasoline users to easily detect adulterated fuel at the point of sales of Petroleum Product, to avoid damage to the combustion engine.
• This method of developing a paper indicator is however efficient and meets the requirements of the issue of detection of gasoline adulteration.

## Graphical abstract

**Figure:**
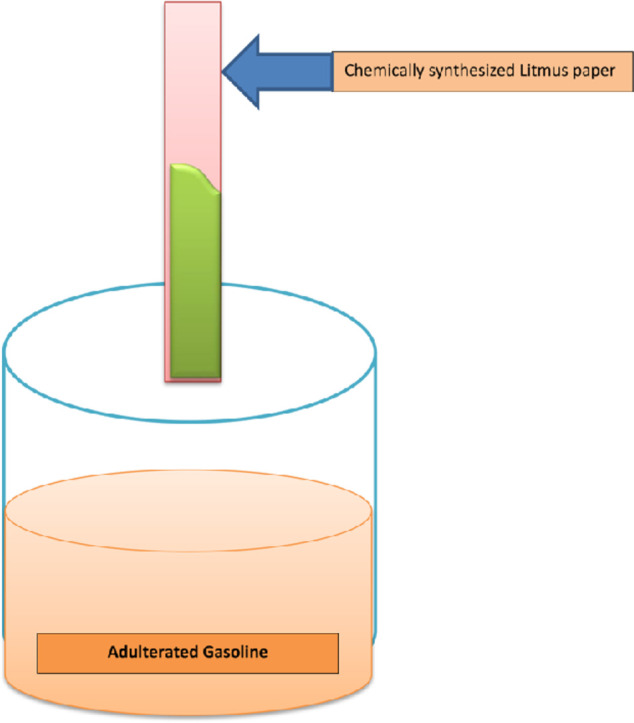

Specifications table

**Table:** 

Subject Area	Energy
More specific subject area	*Petroleum engineering*
Method name	*Fuel Adulteration Detection*
Name and reference of original method	*If applicable, include full bibliographic details of the main reference(s) describing the original method from which the new method was derived.*
Resource availability	*If applicable, include links to resources necessary to reproduce the method (e.g. data, software, hardware, reagent)*

## Method details

Adulteration of petroleum products is an act executed every day by corrupt individuals in developing nations with the goal of boosting profit in their business without taking note of the dangerous impact their activities could have on end users. Adulteration can be defined as the unauthorized or unlawful introduction into gasoline and similar substances of foreign substances resulting in a product's not complying with the requirements and product specifications. It involves deliberate mixing of oil products with' low grade products,' partially refined products or condensates (reservoir gases which, if produced, are condensed to hydrocarbon fluid) with high-demand products such as PMS, DPK, with the singular goal of increasing profit [1], [2], [3], [4].

Foreign substances are also known as adulterants, which in Nigeria are very rampant because fuel adulteration has a very different price because of the introduction of alteration and degradation of the quality of basic transport fuels. This is done by the fuel dealers (adulteration) in order to make the greatest benefit from the product neglecting the motor vehicle damage and any other harmful effect on human life. Fuel adulteration affects many, particularly any vehicle using fuel. The emission of tailpipe increases and then leads to an engine knock. Different products of similar qualities have different prices or end-users do not have efficient tools to identify comparative results of their different characteristics, corrupt sellers will attempt to misuse circumstances in unlawful ways. Illegal practices in the commercial sector are a global surprise, and fuel abuse, together with under-administered goods to end customers, is one of the major malpractices. Fuel adulteration has become an acute problem at point of sale and during transportation [5]. Transport carburant (diesel and gasoline) often become adulterated for monetary gains by other cheaper products or by-products or waste streams of hydrocarbons. The American society for testing materials International (ASTM) has developed some test methods for the detection of adulterated fuel. amongst these methods includes; Density tests, Evaporation tests, Distillation test, Chemical Marker test and Gas chromatography which have all been standardized to detect fuel adulteration. However, none of these methods are termed the most suitable method for adulteration test. The quality of the fuel must be monitored at the distribution point itself to effectively monitor adulteration. The equipment should be portable for this purpose and the measurement method should be rapid and able to provide test results in a very short time. The measuring devices should also be cheap (as a large number of such devices are required) and easy to use. But new methods that are non-toxic, stable and relatively cheap are needed for research projects, in comparison to the other methods developed in the search for a solution in detecting adulterated fuel in Nigeria. Thus, this method has identified using the litmus paper as the most efficient method to detect the increased rate of fuel adulteration in the oil and gas industry of Nigeria.

## Methodology

This method describes the experimental process for detecting fuel adulteration. The research materials and the analytical equipment's used to carry out the experimental procedures were demonstrated. This research consists of two major experiments. These are;

1) The production of paper indicator
2) Using the litmus paper produced, to detect the possible adulterants that are present in the fuels.

The paper indicator is one of the most known affiliate of the chemical indicators. Paper indicators generally involves change of colour when exposed to certain compounds. It helps to reveal the pH level of a sample. The pH level shows us the acidic or basic nature of a substance based on its hydrogen ion concentration or the presence of a particular compound. There are different methodology to the manufacturing of a paper indicator but in this research work, we would employ a basic method.

### Materials description

The material needed for this research includes; beakers or local cup, tap water and distilled water, petrol, ethanol, pH metre, spatula, fume cupboard and filter paper strips.

The Reagents used includes;

Potassium dichromate (Kr_2_Cr_2_O_7_), ethanol, tap water and sulphuric acid.

### The beaker

**Fig. 1:**
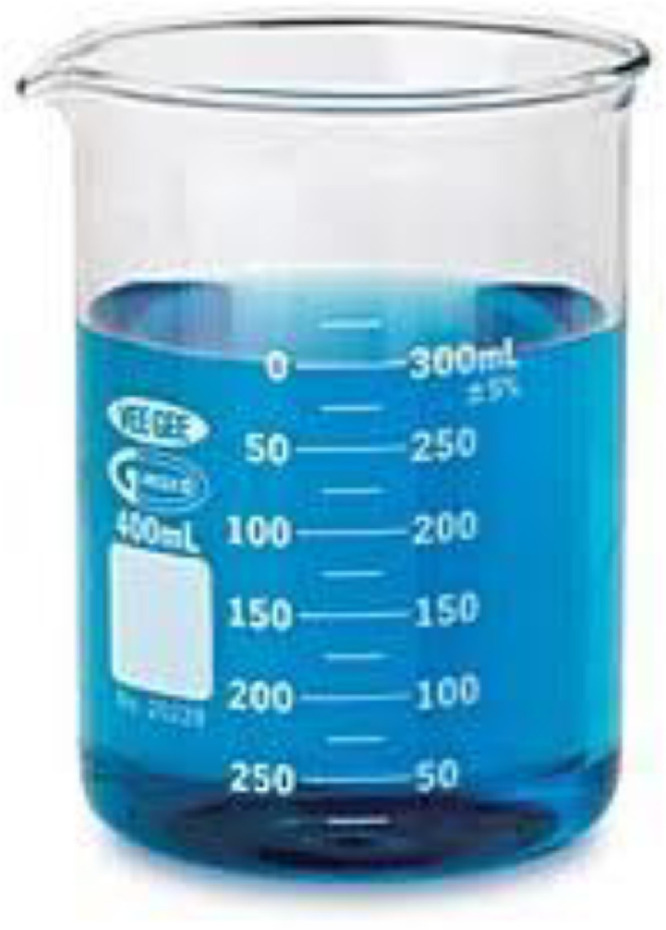
Beaker.

This is a glassware with a flat bottom used to carry solutions as shown in Fig. 1. The beaker is used in the laboratory for storing, mixing and heating liquids. The majority are made of glass, but there are other non-corrosive materials, such as metal and heat-resistant plastics.They are of various sizes. Other apparatus used along with the beakers are Bunsen burners, heat plates, gloves, lab coats etc. The beaker will be used to measure the quantity of each of the compound to be used at their different ratios.

### The fume hood

**Fig. 2:**
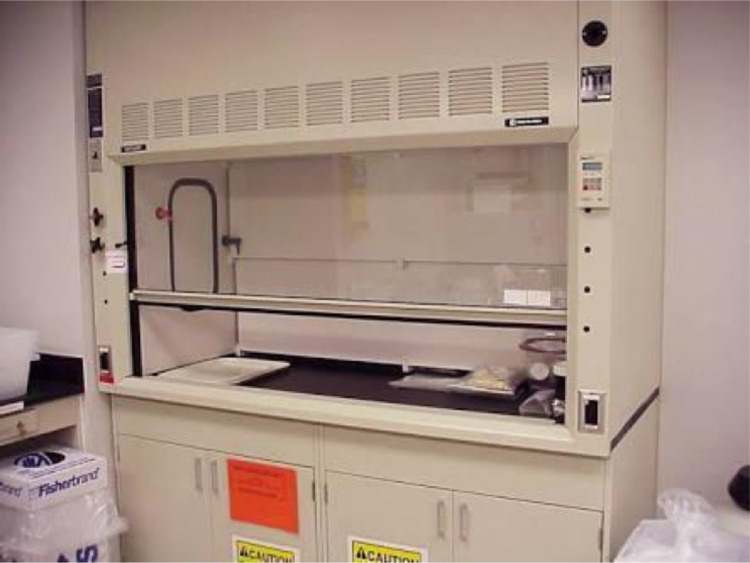
The Fume hood.

The function of a hood is to be a toxic, aggressive or inflammable measure for vapours, gases and aerosols. They are the main method of monitoring laboratory exposures. This apparatus was used because some of the reactions/ reactants used were carcinogenic in nature, so therefore, this was a safety apparatus to use to carry out operations which are highly hazardous to human health. A typical fume cupboard is a cabinet with a moveable front window made out of safety glass as shown in the Fig. 2.

### Spatula

This equipment is a broad and flat flexible blade used to mix, spread and lift materials including foods, drugs, plaster and paints, but in this context, the spatula was used to lift our chemicals in measurable quantities. The spatula varies in different sizes and is basically for the mixing purposes.

### The filter paper

This is a semi- permeable paper. It is basically used to a liquid or air flow. It is used to separate fine substances from liquids or air. It is used in the science laboratories to remove solids from liquids. This can be used to remove sand from water. In this research work, the paper indicator used here was the filter paper because of its permeable qualities.

### Procedures for the experiment

The experimental procedures carried out for this research work is hereby listed below;

- First of all, find an indicator. The adulterant used in this research work is ethanol, therefore there is need to find what will react with ethanol to give a colour change. What we used here is potassium (Kr_2_Cr_2_O_7_) and 1 mol of sulphuric acid
- The filter paper was cut into the required shape and sized as desired. The quantity used was optional.
- The potassium dichloromethane was in solid form and it was converted into a solution by mixing it with 900 ml of water and stir using the spatula in a beaker. Note, this step was done in the fume cupboard because of the corrosive nature of the Kr_2_Cr_2_O_7._
- Next, in the solution formed, add an equi-molar of the sulphuric acid and stir till it mixes.
- The paper strips were prepared and dipped into the solution and leave for about an hour for the mixture to soak into the paper appropriately.
- After an hour, we bring out the paper with a spatula because we need to be careful for the paper not to tear apart because of its soft nature.
- Then leave to dry. The maximum time allowed for the drying process is a maximum of 24 h.
- The litmus paper is now ready to be used.
- To test for its efficiency, dip the paper strip into the adulterated fuel containing the ethanol.
- An obvious colour change in the paper strip is observed.

## Caution

- There is need to wear our PPE (personal protective equipment's) such as the hand gloves, lab coats, nose masks for proper safety during experiment.
- Proper care must be taken for handling these chemicals especially Kr_2_Cr_2_O_7_ because of its corrosive nature to the skin
- Thorough washing of the used equipment and hands with soap and water are high necessary after the experment
- Ensure that you turn off the fume hood after use
- Ensure that all the containers of the chemical used are tightly covered and carefully kept in their respective point of storage.

## Results and Discussion

The use of ethanol for the adulteration of fuel is becoming worldwide. Ethanol is the most produced biofuel in the world, reaching approximately 90 billion litres.

### The solid state of Kr_2_Cr_2_O_7_

This is a compound having a bright orange-reddish crystals and is used in dyeing, staining, bleaching, depolarizer for dry cells. Kr_2_Cr_2_O_7_ is very corrosive and it is also a strong oxidizing agent. It seriously has adverse effect on the human health and should be kept away in isolation far away from human reach.it affects the respiratory tract, human lungs pneumonia, asthma. For it to be in the liquid state, it is usually mixed with water.

### The liquid state of Kr_2_Cr_2_O_7_

In this experiment, the Kr_2_Cr_2_O_7_ was converted into liquid state because it is being infused with the paper indicator. Kr_2_Cr_2_O_7_ is odourless and highly soluble in water. About 4.90 g of the potassium dichromate was dissolved in 1 l of distilled water and about 0.1 N solution was produced. The solid state of Kr_2_Cr_2_O_7_ is carcinogenic in nature, therefore handgloves must be worn to carry out this mixture. This was the second stage of the experiment.

#### The reaction

For there to be a noticeable colour change of the compounds, the reactors and the reagents was combined together so as to check if there could be a visible result or colour change. After liquidifying the Kr_2_Cr_2_O_7_, it was mixed with ethanol in a beaker to observe if there will be any colour change.

#### A positive reaction

The reaction between Kr_2_Cr_2_O_7_ and ethanol was positive. The Kr_2_Cr_2_O_7_ changed colour from an orange-reddish colour to a green colour as shown in the figure below.

**Fig. 3:**
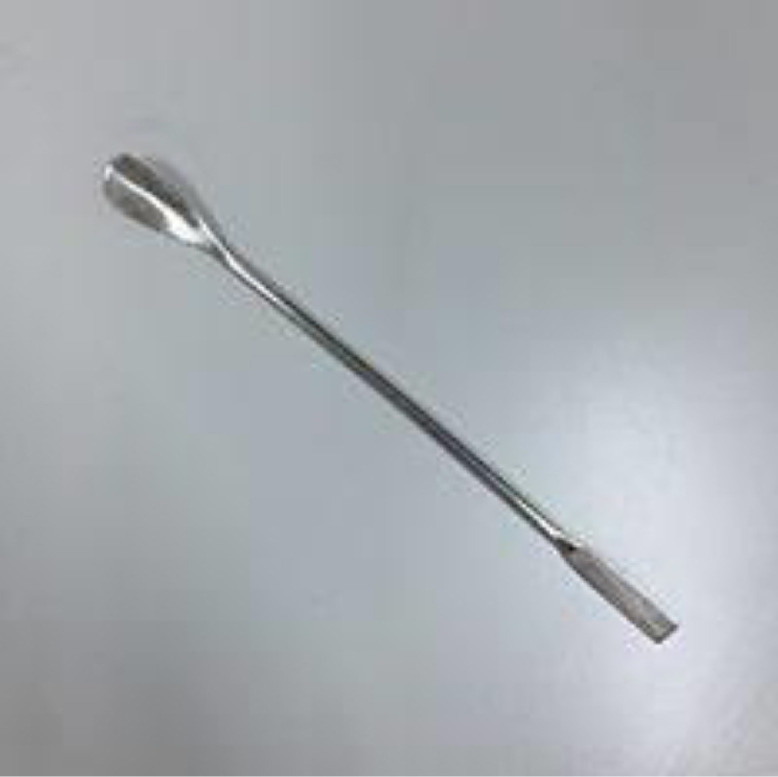
Spatula.

**Fig. 4:**
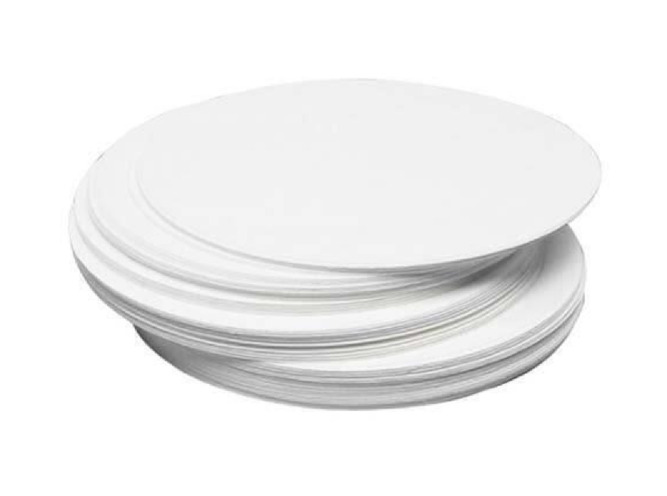
The Filter paper.

**Fig. 5:**
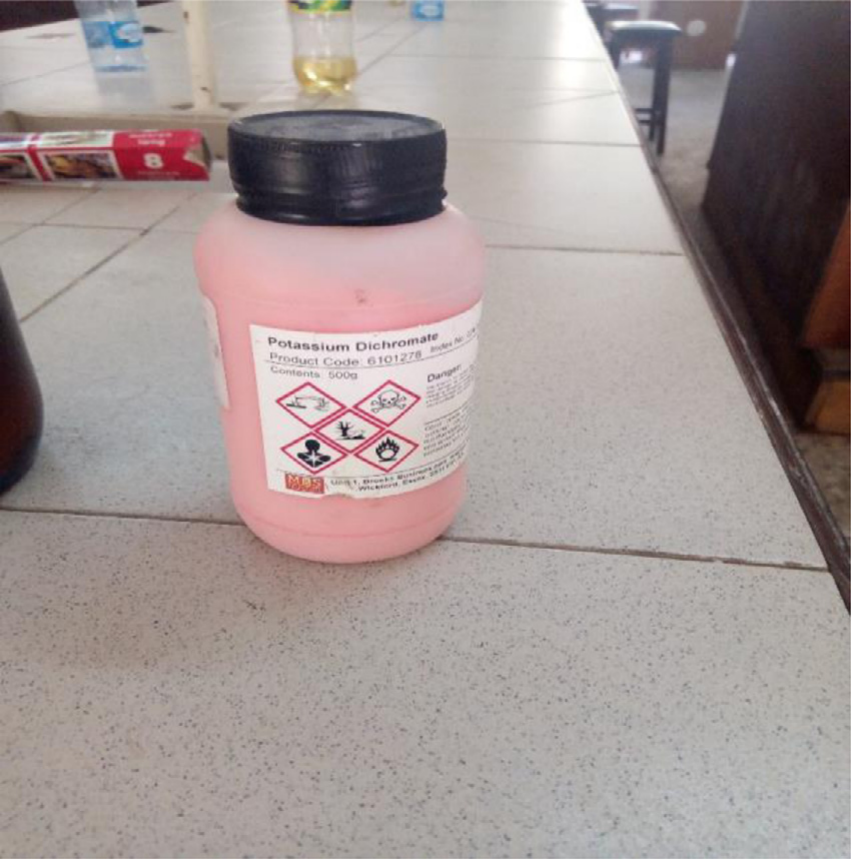
The Solid state of Kr_2_Cr_2_O_7_.

**Fig. 6:**
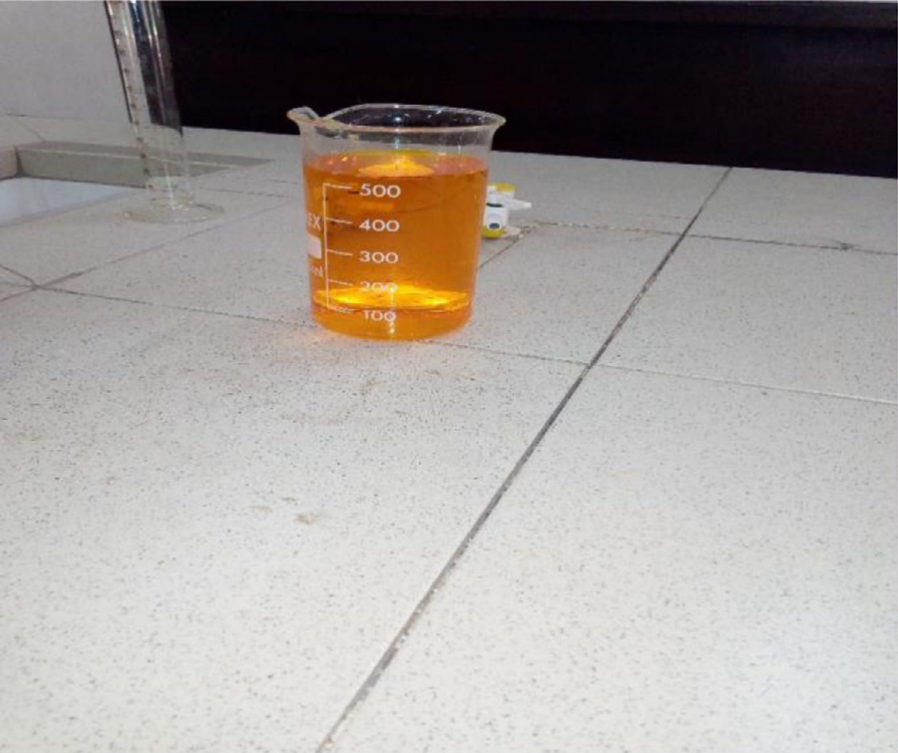
The liquid state of Kr_2_Cr_2_O_7_.

**Fig. 7:**
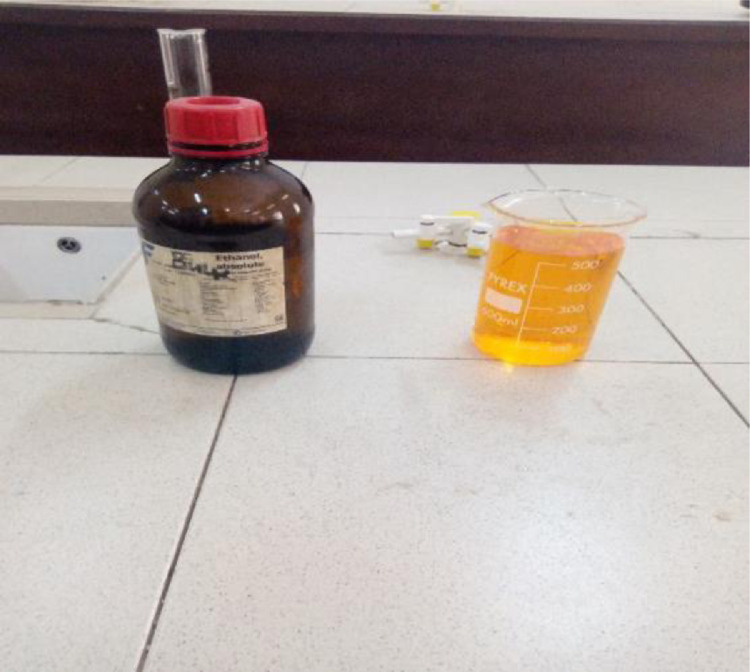
liquefied Kr_2_Cr_2_O_7_ and ethanol.

**Fig. 8:**
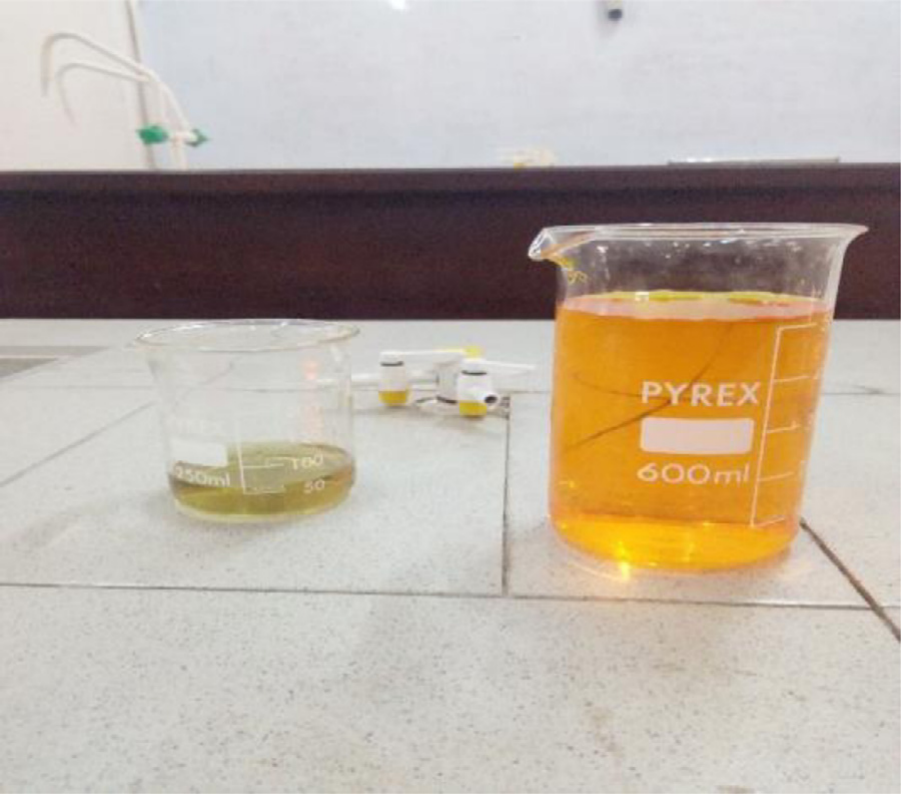
liquefied Kr_2_Cr_2_O_7_ and ethanol.

**Fig. 9:**
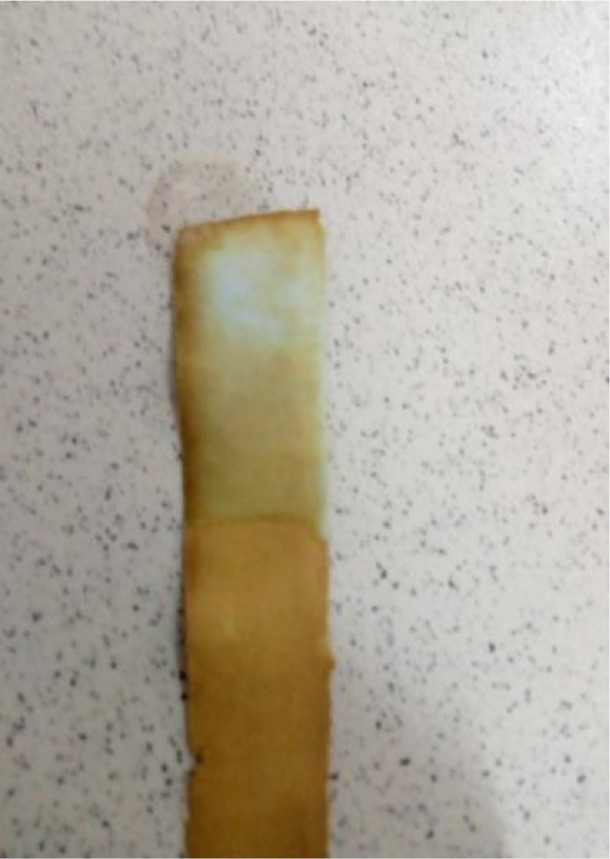
Soaked litmus paper.

Fig. 3, Fig. 4, Fig. 5, Fig. 6, Fig. 7, Fig. 8, Fig. 9

### The paper indicator

The paper was infused with the solution of Kr_2_Cr_2_O_7_ and sulphuric acid. It was left in the solution for about 24 h. After that, it was left to completely dry under air for about 12 h. The drying conditions for the paper was not complicated. It could be readily dried under sunlight or at an adequate room temperature. After the paper was completely dried, it was soaked in the adulterated fuel containing an amount of ethanol.

### Safe handling of the produced litmus paper

Provide adequate ventilation. Always wear protective equipment and wash off any spillage from clothing. Keep cool, dry and away from incompatible materials. Avoid physical damage to containers. Do not repack or return unused portions to original containers. Withdraw only sufficient amounts for immediate use. When handling never eat or drink.

Potassium dichromate derived litmus paper can be destroyed after use by soaking and reacting it with nucleophiles such as water, hydroxyl ions, ammonia, thiols and thiosulfate. The reaction of alkylating agents varies greatly, however, and is influenced by the solubility in the reaction medium. The complete reaction can be facilitated by dissolving the agents in ethanol or similar solvents [6,7]. Dipping and soaking the produced litmus paper in the petrol with or without ethanol less the toxicity of Potassium dichromate derived litmus paper.

As Potassium Dichromate is a powerful oxidising agent [8], [9], the ethanol (alcohol group) will be oxidised, first to the aldehyde (acetaldehyde in this case) and then to the carboxylic acid (acetic acid), The orange dichromate ion in turn is reduced to a green Cr3+ salt after used. The presence of ethanol as an adulterant in the fuel makes the dichromate crystals change from orange to green as the ethanol was oxidised.

### Summary

**Fig. 10:**
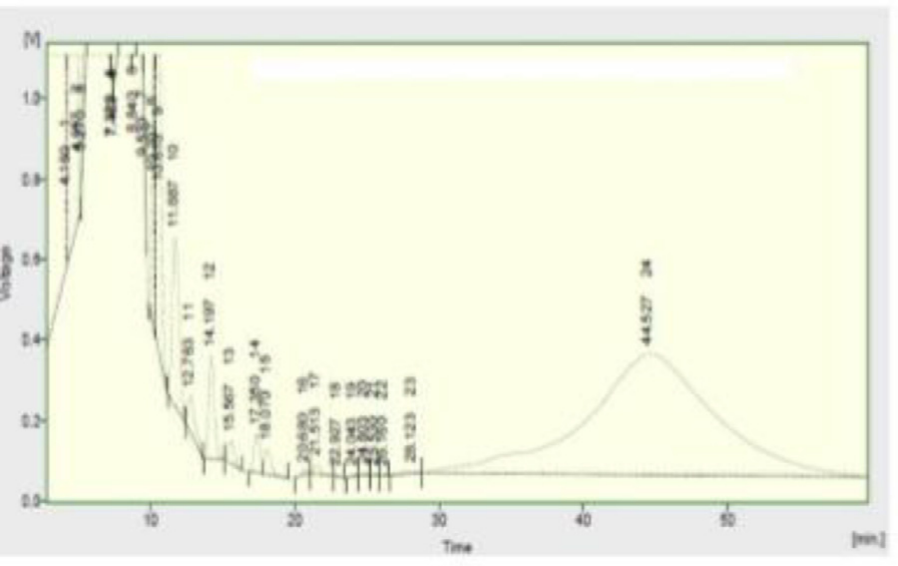
compositions of pure petrol.

Utilization of the fuel adulteration mechanism can help in establishing the maximum operating capacity of the fuels at the given standard operating condition. This is a very necessity in the oil and gas industry because of the rate at which hazards has occurred in the past and it still is presently. Understanding of the essence of this topic is very important especially for correcting the present challenges encountered in the industry as a result of fuel adulteration and hence, prevent future damages. The typical compositions of pure petrol is shown in Fig. 10:

## Conclusions

This project presents the effect of using a paper indicator for the detection of ethanol used as an adulterant in petroleum products (gasoline). For this aim, four core experiments were carried out to investigate the potential of these process. Based on these research work just conducted, the following conclusions were inferred:

- Adulterant in petroleum products are dangerous to consumers who use these products for their vehicles and other places because for instance ethanol mixed with gasoline is far more dangerous than pure gasoline for our vehicles.
- This method of fuel adulteration method is therefore very viable because it is safer, easier and readily available to the oil and gas industry. It can be used directly on field work instead of going through the tasking processes of using other detection methods which are performed in the laboratory.

**Supplementary material *and/or* Additional information:** *[OPTIONAL. We also give you the option to submit both supplementary material and additional information. Supplementary material relates directly to the work that you have submitted and can include extensive excel tables, raw data etc. We would also encourage you to include failed methods or describe adjustments to your methods that did not work. Additional information can include anything else that is not directly related to your method, e.g. more general background information, useful links etc. Introduction is not a section included in the MethodsX format. This information could be moved to the end under Additional Information*.

## Declaration of Competing Interest

The authors declare that they have no known competing financial interests or personal relationships that could have appeared to influence the work reported in this paper.
